# Central and Peripheral Adiposity Had Different Effect on Disability in Centenarians

**DOI:** 10.3389/fendo.2021.635205

**Published:** 2021-03-16

**Authors:** Shanshan Yang, Shengshu Wang, Penggang Tai, Wangping Jia, Ke Han, Miao Liu, Yao He

**Affiliations:** ^1^ Department of Disease Control and Prevention, The ^1st^ Medical Center, Chinese PLA General Hospital, Beijing, China; ^2^ State Key Laboratory of Kidney Disease, Beijing Key Laboratory of Aging and Geriatrics, The 2nd Medical Center, Institute of Geriatrics, Chinese PLA General Hospital, Beijing, China

**Keywords:** centenarians, waist-calf circumference ratio, activities of daily living, instrumental activities of daily living, obesity

## Abstract

**Purpose:**

To explore the correlations between waist circumference, body mass index, calf circumference (CC), and waist-calf circumference ratio (WCR) and activities of daily living (ADLs) and instrumental activities of daily living (IADLs) in Hainan centenarians.

**Patients and Methods:**

A total of 1,002 Hainan centenarians were selected by full sample household survey. ADLs and IADLs were used to investigate the ability of activity and instrumental activity in daily living. The possible non-linear associations were further analyzed using restricted cubic spline.

**Results:**

After adjusting for demographic characteristics (gender, age, ethnicity, marital status, educational level, and type of residence) and lifestyle (smoking, drinking, and exercise), the odds ratio (OR) of CC (continuous variable) on ADL disability in centenarians was 0.90 (95% CI: 0.85–0.96), while high WCR (continuous variable) was related with high risk of ADL disability (OR=1.73; 95% confidence interval[CI], 1.07–2.80). The ORs of CC and WCR for IADL severe disability were 0.86 (95% CI, 0.82–0.91) and 2.23 (95% CI, 1.52–3.28), respectively.

**Conclusion:**

Central (WCR) and peripheral (CC) adiposity had different effects on disability (ADL and IADL) in centenarians. Even in centenarians, maintaining muscle mass (with higher calf circumference) and avoiding central obesity are of positive significance for the prevention of ADL/IADL disability.

## Introduction

Population aging is the inevitable trend accompanied by society and economic development. China has the largest aging population and is one of the fastest aging countries in the world. By the end of 2015, in China, 222 million people were over 60 years old, accounting for 16.1% of the total population, and 143.8 million (10.5%) people were over 65 years old (1). The rapid growth of the older adults is accompanied by significant medical burdens and the needs for long-term care (2, 3). Hence, it is significant for the elderly to keep healthy and to maintain functional independence both for themselves and the whole society. Activities of daily living (ADLs) and instrumental activities of daily living (IADLs) are indexes used to measure functional and instrumental functional capacity (4). Moreover, a number of studies identified several risk factors (such as physical inactivity, depression, smoking, and alcohol consumption) (5–7) of basic function decline (ADL disability) in older adults. However, the correlations between obesity and ADL disability were unclear and inconsistent in elderly population. Specifically, in the last decades, the “obesity paradox” has been prevalent among the elderly older than 80 years and individuals with chronic diseases. The paradox said that inconsistent with the general population, the obese elderly and the obese patients with chronic diseases have better prognosis and lower disability and mortality than those with normal weight/body mass index (BMI) (8, 9). However, some other studies showed that obesity impaired or was not associated with ADLs and IADLs in elderly patients (10, 11).

Similar with the general population, BMI and waist circumference (WC) are usually used as the criteria for obesity in the elderly. However, due to the natural aging progress, it is difficult to measure the height of the elderly, and the accuracy of BMI in the elderly population, specifically the older adults more than 80 years, is difficult to assess. Furthermore, different from abdominal obesity (indicated by WC) and general obesity (indicated by BMI), peripheral adiposity showed the protective effect of type 2 diabetes mellitus, cerebrovascular disease, and premature death (12). However, studies focusing on the effect of peripheral adiposity on ADLs and IADLs in the elderly population are limited.

Furthermore, in recent studies, waist-calf circumference ratio (WCR) was used as an index to assess the disproportion between abdominal fat and leg muscle mass and was proven to be an independent predictor of cardiovascular disease and hepatic steatosis and fibrosis (13, 14).

In this study, we used central (WC, WCR), peripheral (calf circumference), and general (BMI) adiposity indicators to evaluate the obesity status and to analyze the possible correlations between the obesity status and the prevalence of ADL and IADL disability in a cluster sample of centenarians in China.

## Material and Methods

### The China Hainan Centenarian Cohort Study

China Hainan Centenarian Cohort Study (CHCCS) baseline data were used for the present analysis. Details of the methods have been reported elsewhere (15). Briefly, according to the list of centenarians provided by the Hainan Provincial Civil Affairs Department, from June 2014 to December 2016, a full sample household survey was conducted among all centenarians in 16 cities/counties of Hainan Province.

The inclusion criteria was 1) Permanent residents of Hainan, China; 2) Age ≧ 100 and passed the age check; 3) Be capable of consenting and have agreed to participate. The exclusion criteria was 1) Could not be reached; 2) Failed the age check; 3) Did not cooperate with the investigators (15). In the analysis after 2018, due to the sample data was precious, we checked the respondents who were 99 years old (which was excluded firstly) again, and found that 12 of them registered their Lunar birthday on their ID cards. After being revised to their solar birthday, they met the age verification requirements, so they were included in the analysis later. Therefore, a total of 1,002 centenarians were included in the study, response rate was 83.2% (1,002/1,205, contact established 1,473, died before interview 268) with no missing data in main variables. The baseline data of centenarians were collected by household survey, including questionnaire interviews, physical examination, and laboratory blood sample testing. Questionnaire interviews and human body indicators (height, weight, WC, calf circumference, blood pressure) were all measured by trained local Hainan nurses (speaking local language, could communicate with the local centenarians without language barriers).

### Exposure

Obesity indicators included BMI, WC, calf circumference, and WCR. Moreover, body measurement assessing height, weight, WC, and calf circumference was conducted by trained nurses. When measuring, the centenarians were required to take off their shoes, caps, and coats and their personal belongings such as keys and mobile phones. Height measurement was accurate at 0.5 cm, while weight measurement required two consecutive results with an error of less than 0.5 kg. WC and calf circumference measurement were accurate at 0.1 cm (BMI = height/weight^2^, WCR = WC/calf circumference) (16). According to the *Guidelines for Prevention and Control of Overweight and Obesity in Chinese Adults *(17), obese was defined as follows: (1)BMI≥28 kg/m^2^ and (2) WC≥90 cm in men or WC≥85 cm in women.

WCR was firstly used as a index of central obesity in Korean population without an accepted cut-off value for the diagnosis of obesity (13, 14). In this study, it was used as an indicator of central obesity and showed as continuous variables and quintiles variables. Calf circumference is a measure of lower limb dimension, which reflects the degree of obesity of lower limb. Due to the high proportion of muscle in the lower limb, it was usually used to represent the muscle retention level and nutritional status of the elderly (18, 19). In this study, it was used as an indicator of peripheral obesity and showed as continuous variables and quintiles variables.

### Study Outcome

Basic ADL disability was defined using the Barthel index, Barthel index includes 10 items, including 8 items for self-care activities and 2 items for activities ability, with a total score of 100. The scale is evaluated by a dedicated person, and the higher the score, the better the ability of daily living (20). The centenarians who scored 100 were defined as ADL independent, 61–95 with mild ADL disability, 41–60 withmoderate ADL disability, and ≤40 withsevere ADL disability (21). IADL disability was defined using the Lawton index, the Lawton index has 8 items (Ability to use a telephone, shopping, food preparation, housekeeping, laundry, transportation method, medication use, and handling finances), and was designed in 1969 to assess the skills necessary for community life. For each item in IADL, trained investigators (the nurse) confirmed and judged the questionnaire related questions by asking centenarians and their families whether they could call their children by phone, go to the village fair to buy daily necessities independently, take care of the meals at ordinary times, housekeeping, laundry, go to the town/hospital by bus, medication use, or keep his/her own property. Because patients with cognitive or physical disorders lose IADL ability earlier than bath, eating, toilet and other basic ADL ablities, it is very important to evaluate IADL in the diagnosis stage of cognitive, physical or mixed disorders in the elderly (22), and the centenarians who scored 8 were defined as IADL independent, 6–7 with mild IADL disability, 3–5 with moderate IADL disability, and ≤2 with severe IADL disability (21).

### Measurement

Information on demographic characteristics and lifestyle including age, sex, ethnicity (Han, Li, and others), educational level, marital status (married, widowed, and divorced or never married), residential type (living together with families and living alone), smoking (never, former, or current), alcohol use (never, former, or current) and physical activity (low, medium, and high) (15) was collected using aquestionnaire administered by trained nurses. Educational level was assessed and classified into three groups: illiterate (0 year), primary school (1–6 years), and middle school or higher (>6 years).

### Statistical Analysis

The SPSS version 19.0 and R 3.5.2 were used for data interpretation and analysis. The significance level for all tests was set at a two-tailed α value of 0.05. The differences in the means and proportions were evaluated using the Student’s t-test and chi-squared test, respectively. First, in the logistic regression, the obesity indicators (BMI, WC, calf circumference, and WCR) were considered as the continuous variables, and the dependent variables were ADL disability (mild, moderate, and severe), ADL moderate and severe disability, IADL disability (mild, moderate, and severe), and IADL severe disability. Subsequently, the obesity indicators were considered as the quintile variables. The collinearity test was carried out for all variables before they were included in the regression. Restricted cubic splines were used to display and visually test the association between the risk of ADL/IADL disability and the obesity-related indicators (BMI, WC, calf circumference, and WCR). Restricted cubic splines were conducted by R 3.5.2. Statistical analysis was conducted by SPSS version 19.0.

## Results

### Essential Information

Of the 1,002 centenarians, 822 (82.2%) were predominantly women. The mean age was 102.77 (standard deviation,2.75) years. A total of 165 (16.5%) centenarians were independent on ADL, and only 19 (1.9%) centenarians were independent on IADL. In men, the ratios were 29.4% (53) and 4.4% (8), while in women, the ratios were 13.6% (112) and 1.3% (11), respectively. With the increase of WCR quintile, the proportion of ADL and IADL independence decreased, and the proportion of ADL and IADL severe disability increased (**Table 1**).

**Table 1:** Sociodemographic variables of the China Hainan centenarian cohort study participants.

	All (n=1002)	Waist-calf circumference ratio					P
		Q1 (n=200)	Q2 (n=203)	Q3 (n=194)	Q4 (n=199)	Q5 (n=206)	
Mean ± SD							
Age (years)	102.77 ± 2.75	102.85 ± 2.6	103.11 ± 2.85	102.81 ± 2.91	102.32 ± 2.40	102.81 ± 2.82	0.062
Height (cm)	144.52 ± 8.95	148.08 ± 9.17	145.00 ± 8.40	143.36 ± 9.13	144.2 ± 8.41	142.00 ± 8.54	<0.001
Weight (kg)	37.85 ± 7.69	39.88 ± 8.20	37.88 ± 7.90	38.12 ± 7.93	37.26 ± 6.80	36.17 ± 7.12	<0.001
Waist circumference (cm)	75.27 ± 8.79	70.18 ± 7.42	72.62 ± 7.64	76.62 ± 7.50	76.81 ± 8.49	80.06 ± 9.17	<0.001
BMI (kg/m^2^)	18.11 ± 3.22	18.12 ± 2.96	17.98 ± 3.21	18.54 ± 3.36	17.94 ± 3.04	18.01 ± 3.50	0.332
Calf Circumference (cm)	24.73 ± 3.67	27.51 ± 3.07	25.49 ± 2.77	25.15 ± 2.51	23.62 ± 2.59	21.84 ± 2.57	<0.001
WCR	3.08 ± 0.41	2.56 ± 0.13	2.85 ± 0.07	3.05 ± 0.05	3.25 ± 0.07	3.68 ± 0.3	<0.001
N(%)							
Gender							<0.001
Male	180 (18.0)	71 (39.4)	39 (21.7)	27 (15.0)	30 (16.7)	13 (7.2)	
Female	822 (82.0)	129 (15.7)	164 (20.0)	167 (20.3)	169 (20.6)	193 (23.5)	
Ethnic							0.265
Han	883 (88.1)	168 (19.0)	180 (20.4)	178 (20.2)	174 (19.7)	183 (20.7)	
Li	106 (10.6)	26 (24.5)	21 (19.8)	15 (14.2)	22 (20.8)	22 (20.8)	
Others	13 (1.3)	6 (46.2)	2 (15.4)	1 (7.7)	3 (23.1)	1 (7.7)	
Marital status							0.111
Married	100 (10.0)	29 (29.0)	20 (20.0)	12 (12.0)	18 (18.0)	21 (21.0)	
Widowed	836 (83.4)	154 (18.4)	166 (19.9)	173 (20.7)	171 (20.5)	172 (20.6)	
Divorced or never married	66 (6.6)	17 (25.8)	17 (25.8)	9 (13.6)	10 (15.2)	13 (19.7)	
Education level							0.030
Illiterate	915 (91.3)	173 (18.9)	184 (20.1)	179 (19.6)	186 (20.3)	193 (21.1)	
Primary school	67 (6.7)	17 (25.4)	17 (25.4)	12 (17.9)	12 (17.9)	9 (13.4)	
Middle school or higher	20 (2.0)	10 (50.0)	2 (10.0)	3 (15.0)	1 (5.0)	4 (20.0)	
Residential type							0.176
Living together with families	863 (86.1)	163 (18.9)	172 (19.9)	172 (19.9)	173 (20)	183 (21.2)	
Living alone at home	139 (13.9)	37 (26.6)	31 (22.3)	22 (15.8)	26 (18.7)	23 (16.5)	
Body mass index (kg/m^2^)							0.413
Underweight (<18.5)	575 (57.4)	110 (19.1)	116 (20.2)	100 (17.4)	125 (21.7)	124 (21.6)	
Normal (18.5–24.0)	391 (39.0)	82 (21.0)	81 (20.7)	83 (21.2)	69 (17.6)	76 (19.4)	
Overweight (24.0-27.9)	28 (2.8)	8 (28.6)	4 (14.3)	9 (32.1)	3 (10.7)	4 (14.3)	
Obese (≥28.0)	8 (0.8)	0 (0.0)	2 (25.0)	2 (25.0)	2 (25.0)	2 (25.0)	
WC							<0.001
Normal	885 (88.3)	198 (22.4)	197 (22.3)	169 (19.1)	165 (18.6)	156 (17.6)	
Obese	117 (11.7)	2 (1.7)	6 (5.1)	25 (21.4)	34 (29.1)	50 (42.7)	
Smoking status							0.074
Non-smoker	919 (91.7)	173 (18.8)	184 (20.0)	182 (19.8)	186 (20.2)	194 (21.1)	
Former	52 (5.2)	18 (34.6)	13 (25.0)	8 (15.4)	5 (9.6)	8 (15.4)	
Current	31 (3.1)	9 (29.0)	6 (19.4)	4 (12.9)	8 (25.8)	4 (12.9)	
Drinking status							0.639
Non-drinker	872 (87.0)	168 (19.3)	182 (20.9)	167 (19.2)	173 (19.8)	182 (20.9)	
Former	81 (8.1)	17 (21.0)	13 (16.0)	16 (19.8)	18 (22.2)	17 (21.0)	
Current	49 (4.9)	15 (30.6)	8 (16.3)	11 (22.4)	8 (16.3)	7 (14.3)	
Physical activity							<0.001
Low	874 (87.2)	155 (17.7)	176 (20.1)	166 (19.0)	182 (20.8)	195 (22.3)	
Medium	37 (3.7)	14 (37.8)	6 (16.2)	10 (27.0)	2 (5.4)	5 (13.5)	
High	91 (9.1)	31 (34.1)	21 (23.1)	18 (19.8)	15 (16.5)	6 (6.6)	
ADL							<0.001
Independent	165 (16.5)	53 (32.1)	40 (24.2)	29 (17.6)	23 (13.9)	20 (12.1)	
Mild disability	550 (54.9)	122 (22.2)	116 (21.1)	113 (20.5)	101 (18.4)	98 (17.8)	
Moderate disability	142 (14.2)	21 (14.8)	28 (19.7)	22 (15.5)	34 (23.9)	37 (26.1)	
Severe disability	145 (14.5)	4 (2.8)	19 (13.1)	30 (20.7)	41 (28.3)	51 (35.2)	
IADL							<0.001
Independent	19 (1.9)	5 (26.3)	7 (36.8)	2 (10.5)	2 (10.5)	3 (15.8)	
Mild disability	61 (6.1)	20 (32.8)	9 (14.8)	10 (16.4)	10 (16.4)	12 (19.7)	
Moderate disability	274 (27.3)	81 (29.6)	67 (24.5)	50 (18.2)	44 (16.1)	32 (11.7)	
Severe disability	648 (64.7)	94 (14.5)	120 (18.5)	132 (20.4)	143 (22.1)	159 (24.5)	

The distribution of each item of ADL and IADL in the centenarians was showed in **Table S1**. And the performance of male was higher than that of female in each item of IADL (P<0.05, **Table S1**), while there was no significant gender difference in the performance of items of ADL except Stair Climbing, Chair/Bed Transfer, Ambulation and Self-bathing (**Table S1**).

### Association Between Obesity Indicators and ADL Disability

**Table 2** model D shows that, after adjustment, compared with the lower BMI and calf circumference, the possibility of ADL disability decreased by 7% (OR=0.93, 95% confidence interval [CI], 0.88–0.99) for each unit of BMI increase and decreased by 10% (OR=0.90, 95%CI:0.85–0.96) for each centimeter of calf circumference increase. While WCR was found to be associated with ADL disability positively (OR=1.73;95% CI, 1.07–2.80, **Table 2**). The effect of calf circumference was insignificant after being adjusted in men, while it was still significant after being adjusted in women. Moreover, after adjusting the WCR, the coefficient of WCR on ADL disability was significant in men and was insignificant in women (**Table 2**). Furthermore, when the dependent variable resulted in moderate and severe ADL disability, the coefficients of calf circumference and WCR were stronger than the coefficients when the dependent variable was ADL disability. The OR of calf circumference was 0.83 (95% CI, 0.79–0.87) in the whole population, and the ORs were 0.91 (95% CI, 0.81–1.02) in men and 0.81 (95% CI, 0.76–0.85) in women after adjustment. Furthermore, the ORs of WCR were 2.81 (95% CI, 1.95–4.05), 4.61 (95% CI, 1.65–12.94), and 2.67 (95% CI, 1.80–3.98), respectively (**Table 2**).

**Table 2:** The odds ratios for activity of daily (ADL) disability and moderate and severe ADL disability.

	ADL disability				ADL moderate and severe disability			
	Model A	Model B	Model C	Model D	Model A	Model B	Model C	Model D
Male								
BMI	0.95 (0.85–1.07)	0.94 (0.84–1.06)	0.94 (0.84–1.07)	0.94 (0.83–1.07)	1.06 (0.94–1.20)	1.06 (0.94–1.20)	1.07 (0.95–1.22)	1.08 (0.95–1.24)
WC	1.03 (0.99–1.07)	1.03 (0.99–1.07)	1.03 (0.99–1.08)	1.04 (0.99–1.08)	1.04 (1.00–1.08)	1.04 (1.00–1.08)	1.04 (1.00–1.09)	1.04 (1.00–1.09)
CC	0.90 (0.82–1.00)	0.91 (0.82–1.01)	0.92 (0.82–1.02)	0.91 (0.82–1.02)	0.92 (0.83–1.02)	0.92 (0.82–1.02)	0.92 (0.82–1.03)	0.91 (0.81–1.02)
WCR	5.48 (1.92–15.64)	5.25 (1.83–15.08)	5.14 (1.73–15.25)	5.83 (1.89–17.99)	3.50 (1.41–8.68)	3.63 (1.45–9.07)	3.73 (1.43–9.73)	4.61 (1.65–12.94)
Female								
BMI	0.93 (0.88–0.99)	0.93 (0.88–0.99)	0.93 (0.88–0.99)	0.93 (0.88–0.99)	0.94 (0.90–0.99)	0.94 (0.90–0.99)	0.94 (0.90–0.99)	0.95 (0.90–0.99)
WC	0.98 (0.95–1.00)	0.98 (0.96–1.00)	0.98 (0.96–1.00)	0.97 (0.95–0.99)	0.97 (0.95–0.99)	0.97 (0.95–0.99)	0.97 (0.95–0.99)	0.97 (0.95–0.99)
CC	0.88 (0.83–0.94)	0.88 (0.83–0.94)	0.89 (0.83–0.94)	0.91 (0.85–0.97)	0.79 (0.74–0.83)	0.79 (0.74–0.83)	0.79 (0.74–0.84)	0.81 (0.76–0.85)
WCR	1.63 (0.97–2.75)	1.68 (0.99–2.84)	1.65 (0.97–2.79)	1.12 (0.65–1.93)	3.08 (2.10–4.52)	3.10 (2.11–4.54)	3.08 (2.09–4.54)	2.67 (1.80–3.98)
Total								
BMI	0.92 (0.88–0.97)	0.94 (0.89–0.99)	0.93 (0.89–0.99)	0.93 (0.88–0.99)	0.95 (0.91–0.99)	0.96 (0.91–1.00)	0.96 (0.91–1.00)	0.96 (0.92–1.00)
WC	0.98 (0.97–1.00)	0.99 (0.97–1.01)	0.99 (0.97–1.01)	0.99 (0.97–1.01)	0.98 (0.96–1.00)	0.98 (0.97–1.00)	0.98 (0.97–1.00)	0.98 (0.96–1.00)
CC	0.86 (0.82–0.91)	0.89 (0.84–0.94)	0.89 (0.85–0.94)	0.90 (0.85–0.96)	0.82 (0.78–0.86)	0.81 (0.77–0.85)	0.82 (0.77–0.86)	0.83 (0.79–0.87)
WCR	2.70 (1.71–4.26)	2.18 (1.36–3.48)	2.13 (1.33–3.41)	1.73 (1.07–2.80)	3.15 (2.23–4.45)	3.15 (2.21–4.48)	3.10 (2.17–4.43)	2.81 (1.95–4.05)

BMI, body mass index; WC, waist circumference; CC, calf circumference; WCR, waist-calf circumference ratio.

Model A: crude model;

Model B: Adjusted for gender, age;

Model C: Adjusted for gender, age, ethnic, education level, residential type;

Model D: Adjusted for gender, age, ethnic, education level, residential type, smoking, drinking, physical activity.

Furthermore, BMI, WC, calf circumference, and WCR were divided by quintile and considered as categorical variables in the logistic regression models. Compared with Q1, with the increase of calf circumference, the possibility of ADL disability decreased, while with the increase of WCR, the possibility increased, and the trend P value was less than 0.01. The OR of Q5 calf circumference was 0.29 (95%CI, 0.16–0.55), and the OR of Q5 WCR was 1.98 (95%CI, 1.09–3.62) after adjustment (**Table 3**).

**Table 3:** The odds ratios for activity of daily living (ADL) disability and moderate and severe ADL disability (quintile).

	ADL disablity				ADL moderate and severe disability			
	Model A	Model B	Model C	Model D	Model A	Model B	Model C	Model D
BMI								
Q1	1	1	1	1	1	1	1	1
Q2	0.77 (0.44–1.33)	0.77 (0.44–1.35)	0.77 (0.44–1.37)	0.75 (0.42–1.36)	0.99 (0.64–1.51)	0.99 (0.64–1.51)	1.00 (0.65–1.55)	0.99 (0.64–1.54)
Q3	1.07 (0.59–1.92)	1.08 (0.59–1.95)	1.06 (0.58–1.93)	1.15 (0.61–2.14)	1.33 (0.88–2.01)	1.33 (0.88–2.01)	1.32 (0.87–2.01)	1.37 (0.89–2.12)
Q4	0.64 (0.37–1.11)	0.65 (0.37–1.12)	0.66 (0.38–1.15)	0.69 (0.39–1.24)	0.80 (0.51–1.24)	0.80 (0.51–1.24)	0.81 (0.52–1.27)	0.84 (0.53–1.32)
Q5	0.53 (0.31–0.90)	0.52 (0.3–0.89)	0.51 (0.29–0.87)	0.51 (0.29–0.89)	0.64 (0.41–1.01)	0.64 (0.41–1.01)	0.63 (0.40–1.00)	0.65 (0.41–1.04)
WC								
Q1	1	1	1	1	1	1	1	1
Q2	0.85 (0.50–1.45)	0.92 (0.54–1.58)	0.97 (0.57–1.68)	0.77 (0.43–1.35)	1.06 (0.71–1.60)	1.08 (0.71–1.62)	1.12 (0.74–1.69)	0.98 (0.64–1.50)
Q3	0.79 (0.47–1.32)	0.79 (0.47–1.33)	0.83 (0.49–1.41)	0.79 (0.45–1.37)	0.77 (0.50–1.17)	0.76 (0.50–1.16)	0.80 (0.52–1.22)	0.77 (0.49–1.20)
Q4	0.83 (0.50–1.37)	0.84 (0.51–1.40)	0.89 (0.53–1.49)	0.79 (0.46–1.35)	0.78 (0.52–1.16)	0.77 (0.52–1.16)	0.80 (0.53–1.20)	0.74 (0.49–1.13)
Q5	0.85 (0.50–1.44)	0.91 (0.53–1.57)	0.93 (0.54–1.61)	0.79 (0.45–1.41)	0.66 (0.43–1.03)	0.66 (0.43–1.04)	0.67 (0.43–1.05)	0.61 (0.39–0.97)
CC								
Q1	1	1	1	1	1	1	1	1
Q2	0.42 (0.23–0.77)	0.44 (0.24–0.80)	0.45 (0.24–0.83)	0.48 (0.26–0.91)	0.46 (0.32–0.66)	0.45 (0.31–0.66)	0.47 (0.32–0.68)	0.48 (0.33–0.71)
Q3	0.47 (0.23–0.95)	0.49 (0.24–1.00)	0.51 (0.25–1.04)	0.62 (0.30–1.31)	0.29 (0.18–0.47)	0.29 (0.18–0.47)	0.29 (0.18–0.48)	0.32 (0.19–0.53)
Q4	0.26 (0.15–0.48)	0.31 (0.17–0.56)	0.31 (0.17–0.56)	0.38 (0.21–0.71)	0.19 (0.12–0.30)	0.19 (0.12–0.29)	0.19 (0.12–0.29)	0.21 (0.13–0.34)
Q5	0.20 (0.11–0.35)	0.25 (0.14–0.46)	0.26 (0.14–0.49)	0.29 (0.16–0.55)	0.26 (0.16–0.40)	0.25 (0.15–0.39)	0.26 (0.16–0.41)	0.27 (0.17–0.44)
WCR								
Q1	1	1	1	1	1	1	1	1
Q2	1.47 (0.92–2.34)	1.27 (0.79–2.06)	1.24 (0.76–2.01)	1.12 (0.67–1.84)	2.11 (1.24–3.59)	2.12 (1.24–3.62)	2.08 (1.22–3.57)	1.95 (1.12–3.38)
Q3	2.05 (1.24–3.40)	1.74 (1.04–2.93)	1.64 (0.97–2.77)	1.50 (0.87–2.59)	2.56 (1.52–4.34)	2.59 (1.52–4.41)	2.49 (1.45–4.25)	2.39 (1.38–4.15)
Q4	2.76 (1.61–4.72)	2.44 (1.41–4.23)	2.38 (1.37–4.14)	2.03 (1.15–3.61)	4.23 (2.55–7.04)	4.31 (2.57–7.21)	4.22 (2.51–7.08)	3.81 (2.23–6.48)
Q5	3.35 (1.92–5.86)	2.66 (1.49–4.75)	2.60 (1.45–4.65)	1.98 (1.09–3.62)	5.22 (3.16–8.62)	5.30 (3.16–8.87)	5.14 (3.06–8.64)	4.41 (2.59–7.51)

BMI, body mass index; WC, waist circumference; CC, calf circumference; WCR, waist-calf circumference ratio.

Model A: crude model;

Model B: Adjusted for gender, age;

Model C: Adjusted for gender, age, ethnic, education level, residential type;

Model D: Adjusted for gender, age, ethnic, education level, residential type, smoking, drinking, physical activity.

### Association Between Obesity Indicators and IADL Disability

**Table 4** model D shows that, after adjustment, the coefficients of obesity indicators (BMI, WC, calf circumference, and WCR) on IADL disability were insignificant. Similar results were observed in men and women (**Table 4**). Moreover, when the dependent variable resulted in IADL severe disability, the coefficients of calf circumference and WCR were significant in the whole sample and in both men and women. The OR of calf circumference was 0.86 (95% CI, 0.82–0.91) in the whole population, and the ORs were 0.85 (95% CI, 0.76–0.95) in men and 0.87 (95% CI, 0.82–0.92) in women after adjustment. Additionally, the ORs of WCR were 2.23 (95% CI, 1.52–3.28), 3.60 (95% CI, 1.36–9.52), and 2.05 (95% CI, 1.34–3.12), respectively (**Table 4**).

**Table 4:** The odds ratios for instrumental activitity of daily living (IADL) disability and severe IADL disability.

		IADL disability				IADL severe disability			
		Model A	Model B	Model C	Model D	Model A	Model B	Model C	Model D
Male									
BMI		0.91 (0.71–1.17)	0.89 (0.68–1.16)	0.86 (0.65–1.15)	0.85 (0.62–1.17)	0.95 (0.85–1.05)	0.94 (0.84–1.05)	0.94 (0.84–1.05)	0.94 (0.83–1.06)
WC		0.98 (0.90–1.06)	0.97 (0.89–1.06)	0.95 (0.86–1.06)	0.95 (0.85–1.07)	0.99 (0.96–1.03)	0.99 (0.96–1.03)	1.00 (0.96–1.03)	1.00 (0.96–1.04)
CC		0.86 (0.69–1.08)	0.87 (0.69–1.09)	0.88 (0.68–1.15)	0.86 (0.65–1.15)	0.85 (0.77–0.94)	0.86 (0.78–0.95)	0.87 (0.78–0.96)	0.85 (0.76–0.95)
WCR		2.55 (0.3–21.78)	2.16 (0.25–18.41)	1.36 (0.14–13.7)	1.77 (0.13–25.01)	3.19 (1.35–7.52)	2.96 (1.24–7.07)	2.83 (1.14–7.00)	3.60 (1.36–9.52)
Female									
BMI	0.88 (0.76–1.02)		0.88 (0.76–1.02)	0.87 (0.75–1.01)	0.87 (0.75–1.02)	0.94 (0.90–0.98)	0.94 (0.90–0.98)	0.94 (0.90–0.98)	0.94 (0.90–0.99)
WC	0.96 (0.90–1.02)		0.96 (0.90–1.02)	0.96 (0.90–1.02)	0.96 (0.90–1.02)	0.98 (0.97–1.00)	0.98 (0.97–1.00)	0.98 (0.97–1.00)	0.98 (0.96–1.00)
CC	0.87 (0.73–1.04)		0.87 (0.73–1.04)	0.87 (0.72–1.05)	0.88 (0.73–1.06)	0.85 (0.81–0.89)	0.85 (0.81–0.89)	0.85 (0.81–0.90)	0.87 (0.82–0.92)
WCR	1.12 (0.25–4.97)		1.13 (0.25–5.05)	1.07 (0.23–4.88)	0.97 (0.20–4.59)	2.47 (1.67–3.67)	2.58 (1.73–3.84)	2.60 (1.73–3.91)	2.05 (1.34–3.12)
Total									
BMI	0.88 (0.78–0.99)		0.89 (0.78–1.01)	0.88 (0.77–1.01)	0.88 (0.77–1.01)	0.93 (0.89–0.97)	0.94 (0.90–0.98)	0.94 (0.90–0.98)	0.94 (0.90–0.98)
WC	0.96 (0.92–1.01)		0.97 (0.92–1.02)	0.97 (0.92–1.02)	0.96 (0.91–1.02)	0.98 (0.97–1.00)	0.98 (0.97–1.00)	0.99 (0.97–1.00)	0.98 (0.97–1.00)
CC	0.84 (0.74–0.95)		0.87 (0.76–1.00)	0.89 (0.77–1.02)	0.89 (0.77–1.03)	0.84 (0.81–0.88)	0.85 (0.81–0.89)	0.86 (0.82–0.90)	0.86 (0.82–0.91)
WCR	2.20 (0.65–7.42)		1.50 (0.44–5.12)	1.35 (0.39–4.65)	1.25 (0.35–4.39)	2.87 (2.02–4.07)	2.67 (1.86–3.83)	2.66 (1.83–3.85)	2.23 (1.52–3.28)

BMI, body mass index; WC, waist circumference; CC, calf circumference; WCR, waist-calf circumference ratio.

Model A: crude model;

Model B: Adjusted for gender, age;

Model C: Adjusted for gender, age, ethnic, education level, residential type;

Model D: Adjusted for gender, age, ethnic, education level, residential type, smoking, drinking, physical activity.

Furthermore, BMI, WC, calf circumference, and WCR were divided by quintile and considered as the categorical variables in the logistic regression models. Compared with Q1, with the increase of calf circumference, the possibility of IADL severe disability decreased, while with the increase of WCR, the possibility increased, and the trend P value was less than 0.05. The OR of Q5 calf circumference was 0.25 (95%CI, 0.15–0.40), and the OR of Q5 WCR was 2.91 (95%CI, 1.81–4.67) after adjustment (**Table 5**).

**Table 5:** The odds ratios for instrumental activity of daily living severe disability (quintile).

	IADL severe disability			
	Model A	Model B	Model C	Model D
BMI				
Q1	1	1	1	1
Q2	0.57 (0.37–0.86)	0.57 (0.37–0.87)	0.60 (0.39–0.92)	0.56 (0.35–0.89)
Q3	0.98 (0.63–1.51)	0.99 (0.64–1.53)	1.00 (0.64–1.56)	1.05 (0.65–1.70)
Q4	0.60 (0.39–0.91)	0.61 (0.39–0.93)	0.63 (0.41–0.97)	0.65 (0.41–1.03)
Q5	0.51 (0.34–0.78)	0.51 (0.33–0.78)	0.50 (0.33–0.77)	0.48 (0.31–0.76)
WC				
Q1	1	1	1	1
Q2	0.98 (0.65–1.48)	1.04 (0.69–1.59)	1.13 (0.74–1.74)	0.92 (0.59–1.46)
Q3	0.76 (0.51–1.14)	0.78 (0.52–1.17)	0.86 (0.57–1.3)	0.82 (0.52–1.28)
Q4	0.79 (0.54–1.16)	0.81 (0.55–1.19)	0.89 (0.60–1.33)	0.80 (0.52–1.24)
Q5	0.64 (0.42–0.95)	0.68 (0.45–1.02)	0.72 (0.47–1.09)	0.61 (0.39–0.95)
CC				
Q1	1	1	1	1
Q2	0.53 (0.35–0.81)	0.54 (0.35–0.83)	0.55 (0.36–0.85)	0.60 (0.38–0.94)
Q3	0.34 (0.21–0.56)	0.35 (0.21–0.56)	0.36 (0.22–0.58)	0.41 (0.24–0.69)
Q4	0.28 (0.18–0.42)	0.30 (0.19–0.45)	0.29 (0.19–0.45)	0.36 (0.23–0.56)
Q5	0.20 (0.13–0.30)	0.22 (0.14–0.34)	0.23 (0.15–0.37)	0.25 (0.15–0.40)
WCR				
Q1	1	1	1	1
Q2	1.63 (1.10–2.42)	1.52 (1.02–2.27)	1.53 (1.01–2.31)	1.40 (0.90–2.16)
Q3	2.40 (1.59–3.62)	2.26 (1.49–3.44)	2.24 (1.45–3.44)	2.21 (1.40–3.50)
Q4	2.88 (1.9–4.36)	2.83 (1.85–4.33)	2.80 (1.81–4.32)	2.52 (1.59–3.99)
Q5	3.81 (2.49–5.85)	3.51 (2.26–5.46)	3.57 (2.27–5.61)	2.91 (1.81–4.67)

BMI, body mass index; WC, waist circumference; CC, calf circumference; WCR, waist-calf circumference ratio.

Model A: crude model;

Model B: Adjusted for gender, age;

Model C: Adjusted for gender, age, ethnic, education level, residential type;

Model D: Adjusted for gender, age, ethnic, education level, residential type, smoking, drinking, physical activity.

### Non-Linear Correlations

**Figure 1:**
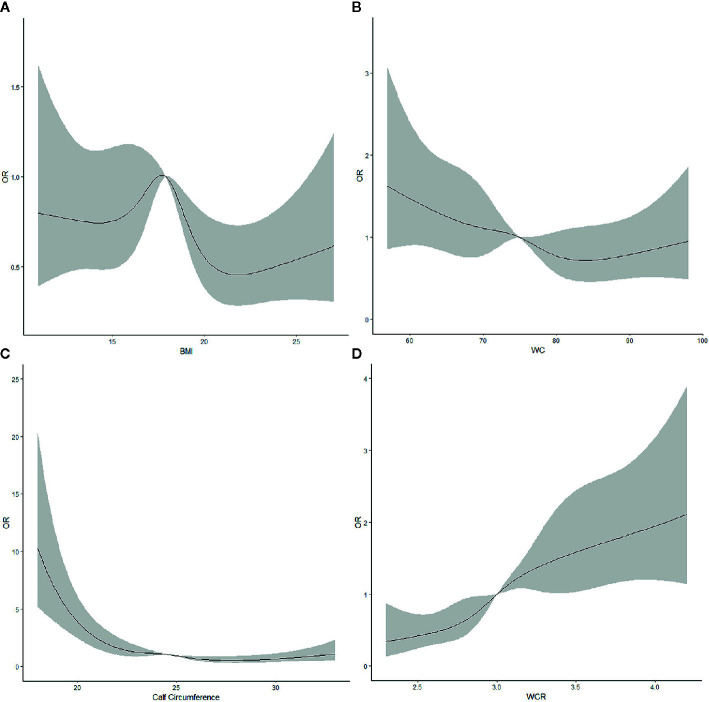
Restricted cubic splines assessing the association between **(A)** body mass index, **(B)** waist circumference, **(C)** calf circumference, and **(D)** waist-calf circumference ratio and the risk of moderate and severe activity of daily living disability after adjusting for gender, age, ethnicity, educational level, residential type, smoking, drinking, and physical activity.

**Figure 2:**
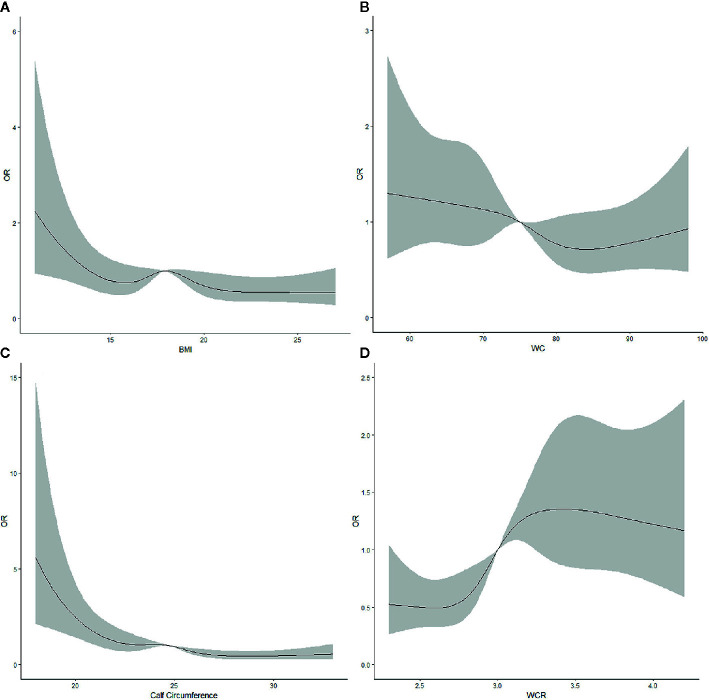
Restricted cubic splines assessing the association between **(A)** body mass index, **(B)** waist circumference, **(C)** calf circumference, and **(D)** waist-calf circumference ratio and the risk of severe instrumental activity of daily living disability after adjusting for gender, age, ethnicity, educational level, residential type, smoking, drinking, and physical activity.

Restricted cubic splines were used to assess the correlations between the prevalence of ADL moderate and severe disability/IADL severe disability and the obesity indicators (WC, BMI, calf circumference, and WCR) (**Figures 1** and **2**). Moreover, the P value of the nonlinear test between calf circumference and ADL moderate and severe disability was 0.0323. The P values of the nonlinear test between calf circumference and IADL severe disability and WCR were 0.0207 and 0.0182, respectively.

## Discussion

Based on the CHCCS baseline data, central (WCR) and peripheral (calf circumference) adiposity had different effects on disability (ADL and IADL) in centenarians. To the best of our knowledge, this is the first study showing the association between WC, BMI, calf circumference, and WCR and ADLs and IADLs in centenarians. The major strength of our study was the comprehensive control and adjustment of a wide range of potential confounders using different statistical models. The similar results demonstrated the robustness of the results. Moreover, as the accuracy of height measurement in the oldest old is difficult to assess, in addition to commonly used indicators such as BMI and WC, in this study, four obesity indicators were included, BMI indicating general obesity, WC and WCR indicating central obesity, and calf circumference indicating peripheral obesity. Calf circumference and WCR were found to have different effects on ADL and IADL disability in centenarians, specifically on ADL moderate and severe disability and IADL severe disability.

The prevalence of ADL independence was 16.5%, and the prevalence of moderate and severe disability ADL was 28.7% according to the CHCCS. This result was similar with that of the Chinese Longitudinal Healthy Longevity Survey (23); the prevalence of no disability in male and women participants aged greater than 100 years old were 7.2% and 18.9%, respectively, and the prevalence of moderate and severe ADL disability was not reported. Moreover, the Newcastle 85+ study (24) showed that the prevalence of ADL disability in elderly individuals aged greater than 85 years was 65.4%, lower than the prevalence in this study, which is possibly associated with the age difference of the two samples. Additionally, the IADL independent rate was 1.9% in this study, and studies regarding IADL of centenarians were limited. A study of participants (aged greater than 65 years) in Spain (25) showed that the ADL independent rate was 46.5%. The low IADL independent rate in Hainan centenarians is associated with the complexity of IADL required, the high rate of illiteracy, and the closed living environment among the centenarians.

WC is usually used to reflect central obesity. Previous studies showed that compared with elderly with normal WC and patients with chronic diseases, elderly and patients with higher WC havebetter prognosis, lower disability, and lower mortality (8, 9). Considering WC as the evaluation criterion of obesity, it is difficult to exclude the impact of nutrition and muscle retention, and the biased conclusion that obesity was beneficial to maintain a better functional capacity (ADL/IADL) was formulated. In this study, when obesity was evaluated by BMI and WC, they showed a tendency to be protectors of ADL/IADL disability, but most of the correlations were insignificant (except in women). However, through the analysis of WCR (the indicator assessing both central obesity and nutrition and muscle retention simultaneously, excluding the effects of nutrition and muscle retention and reflecting the central distribution of fat), WCR was considered a risk factor for ADL/IADL disability in centenarians. WCR was initially used as a central obesity indicator in 3,694 Korean patients with type 2 diabetes and was found to be associated with carotid atherosclerosis (13). To the best of our knowledge, it was initially found to be associated with ADL/IADL disability in this study.

Calf circumference as an indicator of peripheral adiposity was different from the central adiposity, which is mainly due to the fat accumulation in the abdomen that causes the increase of waist circumference, the peripheral adiposity is mainly due to the large proportion of muscle mass that causes the increase of calf circumference (26). Calf circumference is usually used as an indicator of body muscle mass because the legs contain over half of the muscle mass of the body (26). A previous study showed that compared with BMI, calf circumference was a better predictor of nutritional status, functional activity (ADL), and general health conditions in 320 residents living in a nursing home in Central Taiwan (27). Another study of 103 community-dwelling men and women aged 67–92 years showed that lower calf circumference was associated with low ADL and IADL scores (28). Compared with BMI and WC, fewer studies have analyzed the association between calf circumference and ADL/IADL, specifically in centenarians. The calf circumference is a sensitive indicator of decreased activity, specifically in the elderly and in individuals in the state of illness (26). As a protective factor of ADL/IADL disability found in this study, the calf circumference has its reasonable biological basis. It is the indicator of the muscle retention level and nutritional status of the elderly, and in elderly, malnutriton is popular (29), calf circumference may be an indicator variable of nutritional status affecting ADL/IADL disability.

This study has several limitations. First, this study used the baseline data of the CHCCS, which contains cross-sectional data; hence, the effect of causal deduction was not observed. Second, a significant number of centenarians in this study were living in Hainan throughout their lifetime; hence, extra polation of conclusion should be significantly considered. Third, due to the natural aging of the elderly population, there may be bias in height measurement; hence, BMI in this study also has corresponding errors, which may affect the correlation analysis between BMI and ADL/IADL.

Although this study has several limitations, this study has firstly showed the possible correlations between the obesity-related indicators (specifically calf circumference and WCR) and the ADL/IADL in a large sample of Chinese centenarians and found that central (WCR) and peripheral (calf circumference) adiposity had different effects on disability (ADL and IADL) in Chinese centenarians. Even in centenarians, maintaining muscle mass (with higher calf circumference) and avoiding central obesity are of positive significance for the prevention of ADL/IADL disability. Considering that the height of the elderly is difficult to measure and the complexity of WC alone used as an indicator of central obesity, calf circumference and WCR can be used as the measurement indicators of nutrition and obesity, respectively, in the elderly.

## Data Availability Statement

Data are available upon reasonable request from Dr. Yao He; mail: yhe301@x263.net.

## Ethics Statement

The studies involving human participants were reviewed and approved by the CHCCS was approved by the Medical Ethics Committee of the Chinese PLA General Hospital. All participants provided written informed consent for inclusion in the study. The patients/participants provided their written informed consent to participate in this study.

## Author Contributions

SY, ML, and YH contributed to data analysis and manuscript writing. SY, ML, PT, SW, WJ, KH, and YH contributed to study design and data collection. All authors contributed to the article and approved the submitted version.

## Funding

This study was supported by the National Natural Science Foundation of China (81773502, 81703308 and 81703285).

## Conflict of Interest

The authors declare that the research was conducted in the absence of any commercial or financial relationships that could be construed as a potential conflict of interest.
