# Supplementary material for: Central and Peripheral Adiposity Had Different Effect on Disability in Centenarians
Source: Front Endocrinol (Lausanne). 2021 Mar 16;12:635205. doi: 10.3389/fendo.2021.635205 (PMC8008817; doi:10.3389/fendo.2021.635205)
Supplement: Supplementary file 1 [file Table_1.pdf]

Table S1 The distribution of each item of ADL and IADL in the centenarians

N (%)	Total	Male	Female	P
Personal Hygiene				0.109
0	259 (25.8)	38 (21.1)	221 (26.9)	
5	743 (74.2)	142 (78.9)	601 (73.1)	
Self-bathing				0.004
0	326 (32.5)	42 (23.3)	284 (34.5)	
5	676 (67.5)	138 (76.7)	538 (65.5)	
Using the Toilet				0.191
0	142 (14.2)	19 (10.6)	123 (15.0)	
5	251 (25.0)	42 (23.3)	209 (25.4)	
10	609 (60.8)	119 (66.1)	490 (59.6)	
Bowel Control				0.383
0	31 (3.1)	6 (3.3)	25 (3.0)	
5	94 (9.4)	12 (6.7)	82 (10.0)	
10	877 (87.5)	162 (90.0)	715 (87.0)	
Bladder Control				0.356
0	28 (2.8)	5 (2.8)	23 (2.8)	
5	108 (10.8)	14 (7.8)	94 (11.4)	
10	866 (86.4)	161 (89.4)	705 (85.8)	
Getting dressed				0.463
0	98 (9.8)	17 (9.4)	81 (9.9)	
5	199 (19.9)	30 (16.7)	169 (20.6)	
10	705 (70.4)	133 (73.9)	572 (69.6)	
Feeding				0.212
0	58 (5.8)	11 (6.1)	47 (5.7)	
5	216 (21.6)	30 (16.7)	186 (22.6)	
10	728 (72.7)	139 (77.2)	589 (71.7)	
Stair Climbing				<0.001
0	479 (47.8)	60 (33.3)	419 (51.0)	
5	309 (30.8)	61 (33.9)	248 (30.2)	
10	214 (21.4)	59 (32.8)	155 (18.9)	
Chair/Bed Transfer				0.025
0	101 (10.1)	11 (6.1)	90 (10.9)	
5	113 (11.3)	18 (10)	95 (11.6)	
10	375 (37.4)	60 (33.3)	315 (38.3)	
15	413 (41.2)	91 (50.6)	322 (39.2)	
Ambulation				0.007
0	128 (12.8)	14 (7.8)	114 (13.9)	
5	68 (6.8)	6 (3.3)	62 (7.5)	
10	258 (25.7)	44 (24.4)	214 (26)	
15	548 (54.7)	116 (64.4)	432 (52.6)	
Ability to use a telephone				0.015

0	878 (87.6)	148 (82.2)	730 (88.8)	
1	124 (12.4)	32 (17.8)	92 (11.2)	
Housekeeping				0.017
0	661 (66.0)	105 (58.3)	556 (67.6)	
1	341 (34.0)	75 (41.7)	266 (32.4)	
Laundry				0.051
0	528 (52.7)	83 (46.1)	445 (54.1)	
1	474 (47.3)	97 (53.9)	377 (45.9)	
Handling finances				0.001
0	560 (55.9)	80 (44.4)	480 (58.4)	
1	442 (44.1)	100 (55.6)	342 (41.6)	
Food preparation				0.047
0	840 (83.8)	142 (78.9)	698 (84.9)	
1	162 (16.2)	38 (21.1)	124 (15.1)	
Shopping				<0.001
0	907 (90.5)	148 (82.2)	759 (92.3)	
1	95 (9.5)	32 (17.8)	63 (7.7)	
Transportation method				<0.001
0	939 (93.7)	154 (85.6)	785 (95.5)	
1	63 (6.3)	26 (14.4)	37 (4.5)	
Medication use				<0.001
0	701 (70.0)	102 (56.7)	599 (72.9)	
1	301 (30.0)	78 (43.3)	223 (27.1)	
